# Participants Over-Estimate How Helpful They Are in a Two-Player Game Scenario Toward an Artificial Confederate That Discloses a Diagnosis of Autism

**DOI:** 10.3389/fpsyg.2019.01349

**Published:** 2019-06-11

**Authors:** Brett Heasman, Alex Gillespie

**Affiliations:** ^1^Centre for Research in Autism and Education, UCL Institute of Education, London, United Kingdom; ^2^Department of Psychological and Behavioural Science, London School of Economics and Political Science, London, United Kingdom

**Keywords:** online game, diagnostic disclosure, helping bias, positive discrimination, negative discrimination, autism, confederate study, double empathy

## Abstract

Research on how autistic people are perceived by neurotypical people indicates that disclosing a diagnosis leads to a positive discriminatory bias; however, autobiographical autistic accounts indicate that diagnostic disclosure often results in negative discriminatory behavior. We report on an exploratory study to compare people’s self-reported helping behavior with their actual helping behavior toward an assumed autistic collaborator. We led 255 participants to believe that they were interacting online with a real person to play Dyad3D, a maze navigation game where players must work together to open doors, and complete the levels. However, participants were actually playing with an artificial confederate (AC) that is programmed to behave the same way across all interactions. This design enabled us to manipulate the diagnostic status of the AC that participants received prior to collaboration across three conditions: no disclosure, dyslexia-disclosure, and autism-disclosure. We use this method to explore two research questions: (1) is Dyad3D viable in creating a simulated interaction that could deceive participants into believing they were collaborating with another human player online? and (2) what are the effects of disclosing an autism diagnosis on social perception and collaboration? Combined with a post-game questionnaire, we compared differences between diagnostic conditions and differences between self-reported behavior and actual behavior in the game. Our findings show that Dyad3D proved to be an efficient and viable method for creating a believable interaction (deception success rate >96%). Moreover, diagnostic disclosure of autism results in the AC being perceived as more intelligent and useful, but participants also perceived themselves to be more helpful toward the AC than they actually were. We evaluate the strengths and limitations of the current method and provide recommendations for future research. The source code for Dyad3D is freely available (CC-BY-NC 4.0) so that the study is reproducible and open to future adaptation.

## Introduction

There is a growing interest in the role non-autistic people play in shaping social opportunities for autistic people (Milton et al., 2018). While the abilities of autistic people to understand the perspectives of neurotypical others has been well documented, typically developing people have been shown to experience difficulties in interpreting autistic perspectives (Sheppard et al., 2016; Heasman and Gillespie, 2018b; Jaswal and Akhtar, 2018), which can potentially have longer-term consequences for social opportunities for autistic people (Sasson et al., 2017). To date a number of studies have explored how autistic expressions and behavior are perceived by non-autistic people through vignettes and thin-slice judgements (e.g., utilizing video, image, and audio), showing that disclosing a diagnosis of autism significantly improves evaluations (e.g., Chambres et al., 2008; Faso et al., 2014; Brosnan and Mills, 2016). However, reports from autistic people indicate that disclosure of a diagnosis can also result in stigma and negative discrimination (Davidson and Henderson, 2010; Powell and Acker, 2016; Treweek et al., 2018), resulting in a gap in the literature in connecting self-reported perceptions with actual behavior.

We examined the effect of the label “autism” on social perception and behavior on 255 participants through an online collaborative video-game, where the participants believed they were interacting with a human partner to navigate through a maze, when in fact they were interacting with an artificial confederate (AC), programmed to behave the same way for all participants. The impetus for selecting a video-game as a medium for studying interaction was primarily methodological rather than ecological; it provided a controlled environment for manipulating the variable (i.e., the disclosure of a diagnosis), measuring subsequent behavior (i.e., co-operative or uncooperative movements in the game), and repeating the interaction for all participants. In addition to testing the viability of the method, we were curious to see if diagnostic disclosure, even in a primarily goal-orientated logical task, led to any pervasive differences in social perception, and behavior. We therefore examined social perception and behavior of participants in three conditions, (1) a no disclosure condition, (2) a dyslexia-disclosure condition, and (3) an autism-disclosure condition.

### Studies of How Autistic People Are Perceived by Non-autistic People

Research on how autistic people are perceived by non-autistic people has found evidence of both positive and negative discrimination when a diagnosis of autism is disclosed. Using a variety of stimuli (still images, audio, and video), evidence suggests that when no diagnostic information is provided, autistic people are perceived as more socially awkward (Grossman, 2015), idiosyncratic (Brewer et al., 2016), less attractive and less likeable resulting in reduced intention to pursue social interactions (Sasson and Morrison, 2017). These judgments can form very quickly (e.g., based on brief exposure to video: Grossman, 2015) and show little change with increased exposure to stimulus (Sasson et al., 2017). Studies which have manipulated the diagnostic status of stimuli presented to participants have shown that a diagnosis of autism can result in significantly more positive social evaluations (Chambres et al., 2008) and improved affective attitudes (Brosnan and Mills, 2016). However, such effects are shaped by a number of contextual factors, such as the gender (Chambres et al., 2008) and the identity of the perceiver (Gernsbacher et al., 2017), in addition to the medium in which the stimulus is presented (e.g., audio-visual stimulus vs. a speech transcript: Sasson and Morrison, 2017). Moreover, even with knowledge of a diagnosis, ratings have been shown to lag behind those of typically developing targets who were mislabeled as autistic (Sasson and Morrison, 2017). Thus, knowledge of a diagnosis only partially corrects for negative interpretations of autistic behavior and, moreover, positive effects are not always consistently observed.

Autistic reports also indicate that autistic people are misunderstood by non-autistic others resulting in stigma (Dekker, 1999; Chell, 2006; Chown and Beardon, 2017; Treweek et al., 2018). Moreover, media representations have largely focussed on autism as an illness that is a burden to others (Clarke, 2011; Huws and Jones, 2011; Sarrett, 2011; Brownlow et al., 2015), framing autism in terms of a *deficit* rather than a *difference* from a neurotypical majority norm (Smukler, 2005; Ortega, 2009; Kapp et al., 2013; Ridout, 2017). In turn, this has contributed to stigma experienced by autistic people in interpersonal relationships (Hacking, 1999; Heasman and Gillespie, 2018b). There is therefore a gap in terms of connecting the self-reported positive discriminatory behaviors observed in research using vignettes, and the actual behavior of non-autistic people toward autistic people. Yet exploring this perception-behavior gap presents a methodological challenge in terms of establishing a standardized interaction so that comparisons across groups can be observed.

### Methods for Simulating Interactions

Studies of how autistic people are perceived have traditionally used vignettes. Vignettes are passages of text, images, or other types of stimuli (e.g., video) which present a hypothetical situation to participants to elicit a response, either observed, or self-reported (Hughes and Huby, 2004; Grbich, 2013). They are a common technique used in social research to elicit data and can be used to incorporate a variety of detail about social situations; from abstract pictures and short text which impose low cognitive demand, to more elaborate immersive video and audio which draw upon participants’ own experiences (Kinicki et al., 1995). Vignettes can be used to explore automated and intuitive psychological processes, for example Heider and Simmel’s (1944) classic attribution paradigm, where participants observe shapes moving around a scene, and explores how participants impute human social behavior to abstract entities.

Although vignettes provide a controlled way to present context to participants, they remain limited because they do not replicate the pressures of real social life (Hughes and Huby, 2004). In particular, vignettes lack a key social psychological feature involved in human behavior: understanding Self from the social position of Others (Mead, 1934). In vignette studies, participants are not active in the social field they evaluate; rather they evaluate psychological targets knowing that the targets themselves do not perceive them in return. Moreover, while participants passively observe vignettes presented to them, they remain active social agents in the research setting. Thus behavior can be subject to a response bias in light of what participants believe researchers want to observe (Furnham, 1986; McCarney et al., 2007).

It is possible to place participants within an active, yet controlled, social field. Confederate-based studies aim to situate the participant in a controlled social setting where they are interacting with a confederate (i.e., a participant who is actually working for the researcher). This type of design has been classically illustrated, for example through Asch’s (1956) study of conformity, where participants were led by the majority of confederates to report an incorrect answer in judging the lengths of a line, and Milgram’s (1963) study of obedience, where participants were led to believe that they were causing harm to a participant (the confederate) in another room. The advantage of confederate-based studies is that they minimize the impact of potential response biases by focussing more on observed behavior than self-reported data. However, confederate studies require additional methodological considerations, including formalized procedures for interaction, and extensive training to ensure consistency of behavior participants are exposed to (Corti and Gillespie, 2015).

An alternative approach to simulating interactions is through virtual environments, which enable interactions to be replicated while immersing the participant within a social world or scenario (Blascovich et al., 2002; Gillespie et al., 2018). Instead of using the actual presence of others, virtual environments use computer-generated avatars to represent human interactants (Blascovich et al., 2002; Bailenson et al., 2004). Virtual environments allow researchers to decouple rendered behavior from actual behavior through controlling audio-visual perceptual channels (Bailenson et al., 2004; Yee et al., 2009). Moreover, they are adaptable, facilitating research into abstract or more socially complex scenarios. For example, *Cyberball* (Williams et al., 2000) is a virtual ball tossing game used to examine social ostracism, whereby participants are led to believe they are playing with others online, when in truth they play with a virtual agent that is programmed to deliberately exclude the participant from the game. This minimalist format has the advantage of providing a gradable way to manipulate the independent variable of levels of ostracism. Alternatively, virtual environments can encompass a high degree of social complexity (Gillespie et al., 2018), both in terms of the identity that avatars portray, and the communication systems used between avatars (Evans, 2012). “Second Life” is an example of a massively multiplayer online role-playing game (MMORPG) used in psychological research, where users have no task-specific focus other than their own interest in exploring and socializing (Boelstorff, 2008; Evans, 2012). Building on such ideas, we developed *Dyad3D* as a tool that could be used to simulate interactions with participants while controlling an independent variable of diagnostic disclosure.

### Dyad3D Design

To explore how disclosing a diagnosis of autism affects social perception and behavior of non-autistic people, we adapted Heider and Simmel’s (1944) social attribution paradigm, which involves two-dimensional geometric shapes moving around a box, into a three-dimensional computer game. The participant plays using a sphere-shaped avatar which moves around a maze and must work with another sphere-shaped avatar, an AC, that participants believe to be human. Since the AC follows the same path regardless of participants’ actions, it is possible to create the illusion of a collaborative computer-mediated task and thus create a standardized experience of interacting which is the same for all participants.

Combined with a questionnaire administered after the game, this format allows us to examine (1) variation between self-report perception and actual in-game behavior; and (2) variation between groups of participants who have received different labels for the AC. Since we were interested in understanding the effects of disclosing a diagnosis of autism prior to the task, we accordingly grouped participants into an “autism-disclosure” condition (i.e., where participants are led to believe they are playing with an autistic participant) and a control condition (i.e., where no information about a diagnosis is disclosed). We also included an additional group with another diagnosis, dyslexia, to observe whether differences in comparison to the control condition were specific to the label of autism or a diagnostic label in general. Dyslexia was chosen since it is a well-known label to describe difficulties in processing information, there are no associated physical indicators, and like autism it can involve difficulties in planning and organization (Gooch et al., 2011) which are relevant to the nature of the game in which participants must handle multiple tasks (exploring, navigating, and coordinating action).

While advances in graphics and immersive virtual environments provide the opportunity for replicating detailed social situations, we based our study on the minimalist paradigm of Heider and Simmel which uses basic geometric shapes. For practical reasons a minimalist abstract design provided a baseline for testing the viability of the procedure. Further advantages were that using geometric shapes enabled control over different modalities of interaction (e.g., there are no non-verbal interpersonal cues) which meant that differences in perception and behavior could be attributed to the label disclosed and not other social psychological characteristics that have previously been observed to affect interpersonal perception of autistic people (e.g., eye gaze: Sasson et al., 2017). We also did not attempt to replicate any “autistic” behaviors through the geometric shapes. Autism covers such a broad range of abilities and behaviors (Heasman, 2018), and replication would be unrepresentative and potentially uncomplimentary because there are few options to highlight positive autistic behaviors through the simplistic game task. Rather, our intention was to present participants with a misunderstanding within an interaction, since misunderstandings are common across all relationships (Laing et al., 1966) including those involving autistic people (Heasman and Gillespie, 2018b). We wanted to examine how participants made sense of the misunderstanding, and the extent to which they used the partial knowledge of their perceived online partner (i.e., their diagnostic status) to explain their behavior. Moreover, we wanted to observe the potentially pervasive effects of disclosing a diagnosis of autism, in this case whether it would affect participants’ perceptions and behaviors in an extremely abstract and predominantly logical game task. The simplistic use of shapes was also pragmatic: it provides a platform for game development to introduce deeper levels of ecological complexity, thus the source code is freely available to researchers at: https://bitbucket.org/enghoff/dyad3d.

The design process for Dyad3D was iterative over 18 months involving 183 participants, with interviews and focus groups after pilot sessions feeding into further developments of the game. The structure of the game (described with associated pictures below) involves navigating through a maze with an AC that behaves the same way for every participant. Dyad3D ostensibly requires collaboration (hence the name “Dyad”), because some doors in the maze can only be opened by the player, and other doors can only be opened by the AC. However, the game is configured so that the participant progresses successfully in the initial levels before a misunderstanding occurs where the AC deliberately goes the wrong way in the maze and leaves the participant trapped in a prison which severely reduces the participant’s overall score. This perceived “misunderstanding” provides a reference point for participants to discuss and evaluate what went wrong given the partial information they have about their partner. Every participant has the same experience of the interaction unfolding because the AC is programmed to follow a specific path.

Manipulation of the independent variable (i.e., the diagnostic disclosure) is achieved through an option at the start of the game where participants are invited to reflect on their performance in the tutorial of the game through typing a message that is sent to the “online partner.” Diagnostic disclosure is contained in the message participants receive from the AC, with participants randomly assigned to one of three conditions: a no diagnostic disclosure condition, a dyslexia-disclosure condition, and an autism-disclosure condition. Behavior in the game was recorded and a post-game questionnaire examined self-reported perception of the collaboration. The research aims were as follows: (1) to examine whether Dyad3D was viable in creating a simulated interaction that could deceive participants into believing they were collaborating with another player online, and (2) to examine the effects of disclosing an autism diagnosis, both in terms of (2a) comparing self-reported social perception scores with actual behavior in the game, and (2b) examining the qualitative explanations provided by participants about their experience of participation.

## Materials and Methods

### Ethics Statement

BPS and APA procedures regarding informed consent and ethical guidelines were followed, with ethical approval granted by the researcher’s university ethics committee (ref: 000674). Participants were briefed about the nature of the study (i.e., they were informed that they would be navigating through a series of mazes with an online collaborator) and were informed of their right to withdraw at any time. All participants stated in the debrief that they were happy for further participants to experience the same deception and to take part in the study.

### Materials and Measures

#### Dyad3D Game

Dyad3D involves navigating through four mazes of increasing complexity by opening doors to reach a rotating gold cube at the end of each level. The participant plays as a 3-dimensional virtual ball and navigates by using arrow keys on the keyboard. To successfully complete the mazes the participant must work with another ball, the AC, to open a series of doors, and to free each other from a prison at the start of each level. Some doors can be unlocked by the player, and some can be unlocked by the AC.

**FIGURE 1:**
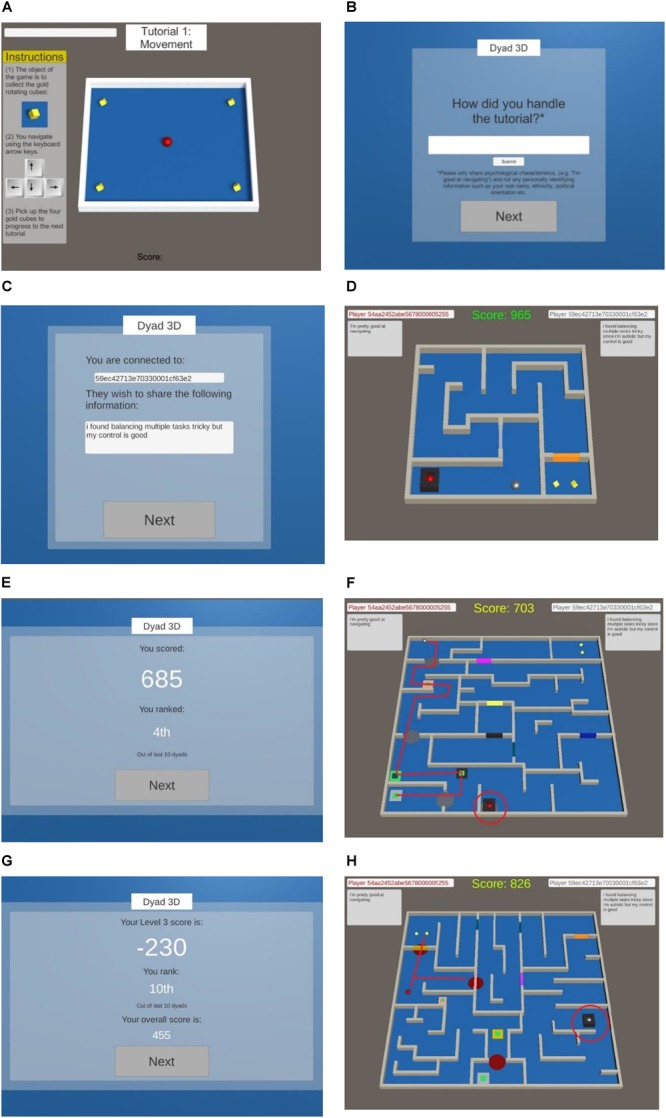
**(A)** Starting Part 1, involving tutorial training for movement. **(B)** Starting Part 2, where participants are invited to reflect on the tutorial and send this information to their online partner (the AC). **(C)** The information received from the AC in the control condition. **(D)** Starting Part 3, where the participant must navigate through the maze. The AC is the silver ball and the human participant is the red ball. **(E)** A leaderboard providing a score and ostensible ranking which is shown after every level. **(F)** Level 3 misunderstanding where the AC, despite immediately unlocking the participant from the prison, chooses instead to take an incorrect route through the maze wasting valuable time. **(G)** The leaderboard after Level 3 which reflects a sharp drop in ranking to last position, 10th. **(H)** Level 4 where the participant is faced with the option of collecting a gold cube before helping the AC, or freeing the AC first.

The game is structured into three parts. In Part 1 the participants completed a tutorial where they were systematically introduced to different elements required to complete a level (e.g., Figure 1A). The tutorial lessons included: (1) navigating to move and “collect” gold cubes by colliding with them; (2) learning how to search for hidden buttons to open doors and collect the gold cubes; (3) learning that some doors can be opened by the red player (participant), and some opened by the silver player (AC), thus collaboration is required; (4) learning how to free the other player from a prison (same process as unlocking doors); and (5) familiarizing with a full game scenario including receiving a score based on time remaining in the level.

In Part 2, participants had the opportunity to reflect on their progress in the tutorial and send a message to their online partner (Figure 1B). Ostensibly, this aimed to aid collaboration by sharing information about strengths and weaknesses. After sharing information, participants were taken to an artificial loading screen and waited for 14 s to be “paired” with another available partner online (in truth, the AC). This was designed to further the illusion that participants were playing with other humans (and thus need to be coordinated) and not directly with an AC. In the next scene, participants were informed they had been successfully paired with a “partner” (Figure 1C). They then received a message from the AC, in which information about the diagnostic status of the AC was manipulated. We chose a statement that offered both positive and negative feedback, indicating difficulty with organizational skills but a strength in navigational ability. For participants in the autism-disclosure condition, the information received was: “I found balancing multiple tasks tricky since I am autistic but my control is good.” For participants in the dyslexia-disclosure condition, the information included “since I am dyslexic” as substitute. Participants in the control condition received: “I found balancing multiple tasks tricky but my control is good.” To further the strength of the deception, fake 24-digit identification numbers were created which matched the style of the participants own anonymous ID (as administered by Prolific). These IDs were displayed at the top of all subsequent screens in the game along with the information shared by the player and AC (Figure 1D).

Part 3 involved playing the game, where participants progressed through four levels of mazes (Figure 1D). At the start of each level the dyad had 1000 points which decreased by eight points a second, with the time stopping when both players picked up their respective gold cubes. In addition, the player and AC alternated in terms of who started the level in a prison and required support from the other to be freed.

The game was designed so that the first two levels were completed very easily and the AC appeared cooperative in terms of efficiently moving and opening doors, freeing the player from the prison, and picking up the gold cube at the end of the level. A fake leaderboard was provided after each level which provided a ranking for performance, ostensibly based on the last ten dyads to complete the game (Figure 1E). The inclusion of the leaderboard was designed to ensure all participants had an equal impression of their collaborative success. In the third level, however, the AC deliberately took the wrong path through the maze and ignored the participant waiting to be freed from the prison (Figure 1F). This negatively impacted the score for the level resulting in a low ranking (Figure 1G).

In the final level of the game, participants were presented with a choice between collecting their own gold cube before freeing their partner from prison (thus reciprocating the experience of themselves being trapped and ignored by the AC in the previous level), or freeing their partner from the prison and then proceeding to collect the gold cube (Figure 1H). This choice was designed to understand behaviorally how participants responded to the misunderstanding that occurred in the prior level.

Since the scores in the game were aggregated for the levels, participants continued to be ranked last (e.g., 10th) after the fourth level despite their efforts. The leaderboard thus provided a benchmark about the severity of the misunderstanding experienced in the third level. Each participant therefore experienced the same ranking of performance: after level 1, they were ranked 4th out of the last ten dyads to register a score; level 2, 3^rd^; level 3, 10^th^; and level 4, 10th.

#### Behavioral Measures

Dyad3D records 11 variables of user input (Table 1). It records the score; calculates mean distance between the player and AC for each level (spatial proximity); mean time difference between the player and AC collecting their respective gold cubes at the end of the level (cube coordination); and mean keystrokes by the user for each level. In addition, it also calculates the number of keystrokes made by the user when they are trapped in the prison on the third level and ignored by the AC (measure of frustration), and the mean duration of each keystroke in time. In the fourth level, it records whether participants free their partner from prison before collecting their own gold cube (termed altruistic behavior), or whether they collect the gold cube before freeing their partner from prison (termed selfish behavior).

**Table 1:** List of behavioral measures from the Dyad3D game.

Measure	Levels	Description
Points score	1–4	A score calculated for the tutorial and for each level in the game. 8 points = 1 s. Each level starts with 1000 points and reduces continually into minus figures
Mean distance (spatial proximity)	1–4	The average distance between the Player and the AC for each level. Measured after the Player or AC are freed from prison and thus working together to complete the level. Unit measurement based on Player diameter
Cube coordination	1–4	Calculates the time difference between the Player and the AC collecting their respective gold cubes
Leadership	1–4	Identifies who picks up the gold cube first, the Player or the AC
Keystroke count	1–4	Counts the number of times the Player hits an arrow button during the levels of the game
Mean keystroke duration	1–4	Calculates the mean duration a key is held down during the game
Bump count	1–4	Counts the number of times the Player and the AC make contact for each level
Prison keystroke count	3	Calculates the number of times the Player presses a keyboard button when trapped in the prison during the 3rd level misunderstanding
Mean prison keystroke duration	3	Calculates the mean duration a key is held down while the payer is trapped in the prison during the 3rd level misunderstanding
Priorities (prosocial or selfish)	4	Identifies whether the Player chooses to free the AC from prison first before collecting a gold cube (prosocial behavior) or whether they choose to collect their own gold cube before freeing the Player
Response time	4	Measures the time between the Player unlocking the AC from prison to when they actually free the AC from prison

#### Questionnaire

Qualtrics was used to administer a post-game questionnaire for participants. Given the priority of first impressions in forming judgements about autistic people (Sasson and Morrison, 2017), we asked participants if they found the information supplied by their partner as useful through a closed-ended question. We further invited participants to explain why they found the information useful or not useful. We also asked participants whether they believed the information provided by their partner affected their own behavior in the game to understand participants’ perceptions about the relationship between diagnostic disclosure and its impact on their behavior in the game.

Additionally, we wanted to understand if there were differences in how participants explained the interaction, thus an open-ended question invited comments on the following points: (1) what worked well in your collaboration? (e.g., useful information shared, hunting for hidden buttons, freeing from prisons, opening doors, deciding which route to take, and dividing up search areas); (2) What could have been improved in your collaboration? (3) Is there anything you could have done differently to support your partner? (4) Is there anything your partner could have done differently to support you? (5) What impressions do you think your partner has of you through their experience of playing the game?

#### The Interpersonal Perception Method

In addition to exploring the effects of diagnostic disclosure, we included rating scales to explore additional perspectives on the task, since social interactions typically comprise multiple perspectives on Self, perspectives on Other, and perspectives on how one is being perceived by others (Mead, 1934; Ichheiser, 1943; Heasman and Gillespie, 2018b). The Interpersonal Perception Method is a way of systematically analyzing the relations between these perspectives (Laing et al., 1966) and has most typically been methodologically operationalized into rating scales (Kenny, 1988; Gillespie et al., 2010; Moore and Gillespie, 2014).

When identifying items to rate we considered criteria from previous studies of social perception of autism. However, we were also limited by the nature of the interaction through the video-game where there is no facial or auditory dimension of engagement. Chambres et al. (2008) used three evaluative dimensions of cognitive, social and emotional items for assessing vignettes of a 6-year-old autistic child’s behavior that participants were either informed or uninformed about their diagnosis. We accordingly included items of “intelligence” (cognitive), “helpfulness” (social), and “frustration” (emotional). We also included “skill,” since the game is dependent on the ability to interact with the computer which participants may feel was a critical factor in the collaboration.

### Participants

A total of 183 participants took part in the pilot study phase to help iteratively develop the game. Participants for the pilots were sourced from the participant pool of the research lab belonging to the researchers’ university. Since the pilot phase exhausted available participants in the participant pool, the full study sourced participants online through a paid participant service provided by Prolific.

A total of 347 participants took part in the full study online. All participants were recruited via Prolific and were paid £5 for 30 min participation. To ensure sample validity through the online recruitment process, we used multiple inclusion/exclusion criteria. First, a demographic restriction was applied given the cross-cultural variation in identifying and understanding autism (Mandy et al., 2014; Obeid et al., 2015). We therefore recruited participants who had English as a first language. Following recruitment, additional inclusion/exclusion criteria were applied. We included an attention check by embedding a closed-ended question within the post-game rating scales that had to be false (“were you unable to finish the game with your partner?”). Participants who failed the attention check were removed from the sample (*n* = 42). A manipulation check was also included to see if participants were aware of the information provided. Those that claimed they did not receive any information or that they did not remember the information shared (*n* = 23) were removed from the analysis. We conducted a deception check through analyzing the free text provided by participants in response to a question which asked them to explain their experience of the study. Participants who mentioned the belief that they were playing with a computer and not a human were excluded (*n* = 9).

Additional criteria for exclusion included participants who: did not complete the game or the questionnaire (*n* = 5), who copied and pasted unintelligible text for open-ended questions (*n* = 3), rated artificially (e.g., the same score for all items in the questionnaire) (*n* = 5), and had technical problems during the study (*n* = 5). In total 92 participants met the exclusion criteria, with 255 participants included in the study, to which all results and findings relate.

Participants were randomly assigned through the survey software Qualtrics to one of three conditions: (1) a control condition (where no diagnostic information was disclosed); (2) a dyslexia-disclosure condition (where a dyslexia diagnosis was disclosed); and (3) an autism-disclosure condition (where an autism diagnosis was disclosed). In the debrief, participants reported that they were happy for other participants to go through the same process, while two participants voluntarily contacted the researchers after the study to express their enjoyment of playing the game, and their surprise that they were playing with an AC and not a human.

### Method of Analysis

To explore RQ1 (how viable is Dyad3D in creating a simulated interaction that could deceive participants into believing they were collaborating with another player online?), we asked participants in the post-game questionnaire to rate the quality of the deception on a six-point scale, from not believable at all (=0) to very believable (=5). Additional checks included: (1) the qualitative responses provided by participants were examined to see if any reference was made to the AC which questioned whether it was actually human; (2) we examined whether attributions of intentionality to the AC were made by participants; (3) participants were asked in the debrief whether they would consent to other participants taking part in the study; and (4) we categorized feedback volunteered by participants who contacted the lead researcher after the study was complete.

To address RQ2a (comparing self-reported social perception scores with actual behavior in the game) one-way ANOVAs were run to explore the effect of condition (no disclosure vs. dyslexia-disclosure vs. autism-disclosure) on survey responses and behavioral data from the game. For ordinal data, non-parametric Kruskal-Wallis one-way ANOVAs were used. Where significant effects were observed, *post hoc* tests with Bonferroni correction examined specific differences between conditions.

To address RQ2b (examining the qualitative explanations provided by participants to understand the role of diagnostic disclosure on their experience of participation) we analyzed participants’ text responses. Specifically, we analyzed participants’ statements about why the information supplied by the AC was useful or not useful with a process of iterative coding (Neale, 2016). Iterative coding involves open-coding participants’ responses, before sorting codes into categories based on the links between codes (Heasman and Gillespie, 2018b). Four main categories resulted from this process, e.g., the category of “tolerance” was formed from statements where participants said the AC information led to lower expectations, higher confidence, greater patience, and increased empathy. The category of “redundant” emerged from statements about the shortcomings of the AC, since some participants deemed the information shared as superfluous, inaccurate, unhelpful, and misleading. The category of “explained misunderstanding” covered statements that specifically linked the AC information to the misunderstanding experienced in the third level of the game (thus no iteration required). Finally, the category of “ambiguous” included statements that were merely descriptive (e.g., “he said how he did the tutorial”), provided tangential information (e.g., “we couldn’t pick the color”), and statements which did not provide meaningful context, (e.g., “yes it did”).

## Results

Table 2 highlights that there were no statistically significant associations between groups in terms of gender χ(4) = 0.729, *p* = 0.948, nationality χ(4) = 0.260, *p* = 0.992, gaming experience χ(4) = 6.09, *p* = 0.193, or ratio of group disclosing a diagnosis χ(2) = 2.27, *p* = 0.321. A one-way ANOVA further investigated the effect of gaming experience on performance in the game, finding no significant association between mean time for completing the tutorial between groups, [*F*(2,252) = 0.310, *p* = 0.734], suggesting participants were of comparable ability to play the game.

**Table 2:** Participant details.

	Control	Dyslexia	Autism	χ^2^	*p*
	(*n* = 80)	(*n* = 83)	(*n* = 92)		
**Gender**				0.729	0.948
Female	45	45	47		
Male	34	36	43		
Unspecified	1	2	2		
**Nationality**				0.260	0.992
United Kingdom	50	52	58		
United States	25	23	27		
Other	5	8	7		
**Diagnoses disclosed by participants^1^**				2.27	0.321
Depression	4	3	1		
Anxiety	4	6	2		
Autism		1	1		
OCD		0	1		
Epilepsy		1	0		
PTSD			1		
Borderline personality disorder			1		
Chronic fatigue syndrome		1			
**Gaming experience**				6.085	0.193
Experienced	30	41	32		
Intermediate	36	34	49		
Novice	14	8	11		

*^*1*^Significance measured as difference in proportion of participants with a diagnosis in each group.*

### RQ1: How Viable Is Dyad3D in Creating a Believable Interaction?

Ratings of the quality of the deception were strong with an overall mean score of 3.83 on a 0–5 Likert scale (0 = not believable at all; 5 = extremely believable), and a one-way ANOVA showed no significant difference between groups [*F*(2,252) = 0.066, *p* = 0.936]. Nine participants believed they were playing with a computer and not a human representing <4% of participants who had passed all other inclusion/exclusion criteria. Moreover, all participants made attributions of intentionality to the AC, as shown by references to the AC’s mental states, emotions and skill/experience, and showing their assumption of human traits. All participants also indicated that they would consent to others taking part in the study, thus the nature of the deception did not result in significant discomfort for participants. Taken together, these data indicate that Dyad3D was predominantly successful in creating a believable interaction, but there is still scope for improvement (see section “Discussion”).

### RQ2a: Differences Between Self- Reported and Behavioral Measures

Table 3 summarizes the differences between groups on self-reported and behavior measures. Kruskal-Wallis *H* tests showed a statistically significant difference between groups regarding the extent to which information provided by the collaborator was perceived as useful, H(2) = 12.74, *p* < 0.002, with a mean rank score higher for the autism-disclosure (autism_usefulness_of_info_ = 140.81) and dyslexia-disclosure (dyslexia_usefulness_of_info_ = 133.38) groups than for the control (control_usefulness_of_info_ = 107.69) group. *Post hoc* pairwise comparisons showed a significant difference between the control and autism-disclosure groups (*p* = 0.002) and the control and dyslexia groups (*p* = 0.028). The results suggest disclosing a diagnosis significantly increased the extent to which participants found the information supplied by the AC as useful.

**Table 3:** Kruskall-Wallis comparison between groups on self-reported and behavioral measures.

						*Post hoc* pairwise		
	df	Mean rank			H	comparisons (Adj Sig.)		
			Dyslexia-	Autism-		Control-	Control-	Autism-
		Control	disclosure	disclosure		autism	dyslexia	dyslexia
Was AC information seen as useful?	2	107.69	133.38	140.81	12.74*	0.020^∗^	0.028^∗^	1.00
Did AC information result in increased self-reported helpfulness?		113.81	131.36	135.96	8.02*	0.020^∗^	0.109	1.00
Did participant prioritize partner over Self?		138.59	117.88	129.01	5.13			
Desire to collaborate with the AC again?		119.23	127.98	134.27	3.12			
Rating AC intelligence		111.61	128.60	140.33	7.45*	0.019^∗^	0.351	0.788
Rating AC skill		125.35	134.02	123.55	1.08			
Rating AC helpfulness		126.06	126.90	129.29	0.10			
Rating AC frustration		131.79	124.16	126.74	0.48			
Rating dyad spatial coordination		133.03	121.10	128.40	1.15			
Rating dyad calmness		128.06	125.23	129.03	0.13			
Rating dyad efficiency		124.60	128.18	129.41	0.20			
Rating dyad understanding		128.58	126.50	127.45	0.03			
Rating self intelligence		136.13	122.26	124.67	2.00			
Rating self skill		131.50	126.68	124.75	0.41			
Rating self helpfulness		141.24	119.79	122.43	4.58			
Rating self frustration		132.77	126.75	123.59	0.71			

*^∗^*p* < 0.05.*

There was a significant difference between groups in reporting perceived helpfulness toward the AC because of the information shared, H(2) = 8.02, *p* = 0.018, with a mean rank score higher for the autism-disclosure (autism_info_affected_helpfulness_ = 135.96) and dyslexia-disclosure (dyslexia_info_affected_helpfulness_ = 131.36) groups than for the control (control_info_affected_helpfulness_ = 113.81) group. *Post hoc* pairwise comparison with Bonferroni correction showed a significant difference between the control and autism-disclosure groups (*p* = 0.020) but not the control and dyslexia-disclosure groups (*p* = 0.109). The results suggest disclosing a diagnosis increased the extent to which participants perceived they acted more helpfully during the game, but only significantly for the autism-disclosure and not the dyslexia-disclosure group.

However, although participants in the autism-disclosure group showed a greater tendency to prioritize their partner’s interest (freeing their partner from prison before collecting their own gold cube) than prioritizing their own interests (picking up the gold cube before releasing their partner from prison) compared with the control and dyslexia-disclosure group (mean ranks: control_priorities_ = 138.59; autism_priorities_ = 117.88; dyslexia_priorities_ = 129.01), this difference was not significant H(2) = 5.13, *p* = 0.077. Further Chi-square comparisons showed no significant association between perceiving oneself to be helpful with actual helping behavior χ(1) = 0.185, *p* = 0.667. These findings compare with parametric one-way ANOVAs which found no significant differences between groups for mean time to complete levels [*F*(2,252) = 0.811, *p* = 0.446], mean spatial proximity between the participant and their other player across the levels [*F*(2,252) = 0.654, *p* = 0.521], or mean frustration (measured as the mean time participants hold down a keyboard key while trapped in the prison), [*F*(2,243) = 2.770, *p* = 0.65]. These results suggest that despite participants in the autism-disclosure group believing they were more helpful compared with participants in the control group, they did not significantly differ from the control group when it came to actual helping behavior in the game.

There was a significant difference between groups in participants rating AC intelligence, [H(2) = 7.452, *p* = 0.024] with participants in the autism-disclosure group rating their partners higher than the control or dyslexia-disclosure group (mean rank scores: autism_intelligence_other_ = 140.33 control_intelligence_other_ = 111.61 dyslexia_intelligence_other_ = 128.60). *Post hoc* pairwise comparison with Bonferroni correction showed a significant difference between the control and autism-disclosure groups (*p* = 0.019), but no significant difference between the control and dyslexia-disclosure groups (*p* = 0.788). This result was consistent when analyzing the differences between rating one’s own intelligence and rating their partner’s intelligence [H(2) = 8.327, *p* = 0.016], with *post hoc* pairwise comparison showing significant differences between the autism-disclosure and control groups (*p* = 0.023). The results suggest a difference in effect between diagnostic labels, with a disclosure of autism leading to significantly higher perceptions of intelligence than a disclosure of dyslexia when compared with the control group.

Criteria where no significant differences between groups were observed include: participants rating the AC’s skill [H(2) = 1.080, *p* = 0.583], helpfulness [H(2) = 0.097, *p* = 0.953], and frustration [H(2) = 0.475, *p* = 0.789]; perceived ratings by the AC in terms of intelligence [H(2) = 5.605, *p* = 0.61], skill [H(2) = 0.407, *p* = 0.816], helpfulness [H(2) = 1.151, *p* = 0.563] and frustration [H(2) = 0.568, *p* = 0.753]; perceptions of teamwork in terms of calmness [H(2) = 1.363, *p* = 0.506], efficiency [H(2) = 0.845, *p* = 0.655], or understanding [H(2) = 0.285, *p* = 0.867]; desire to collaborate again with the AC [H(2) = 3.12, *p* = 0.210]; and participants rating their own intelligence [H(2) = 2.002, *p* = 0.368], skill [H(2) = 0.406, *p* = 0.816], helpfulness [H(2) = 4.584, *p* = 0.101] or frustration [H(2) = 0.705, *p* = 0.703]. Taken together, these results indicate that disclosing a diagnosis results in positive discrimination in terms of higher perceptions of intelligence, finding information provided by the AC as more useful and resulting in greater tolerance, and more positive perceptions of being helpful toward the AC. However, in each case these effects were significant for the autism -disclosure group in comparison to the control group, but not so for the dyslexia-disclosure group (where participants were only significantly different from the control group in terms of finding the information provided by the AC as more useful). This suggests that positive discrimination observed due to the disclosure of an autism diagnosis is specific to autism and not the wider presence of a label in general.

### RQ2b: Explanations Provided by Participants About Their Perception and Behavior Toward the AC

Explanations provided by participants about the utility of the AC information was categorized into three types (see Table 4), which accounted for 80% of all participants. These categories included: (1) participants who felt that the information supplied was redundant or inaccurate and of no use to facilitating the game collaboration (termed “information redundant”); (2) participants who claimed that the information provided helped them to make sense of why they were left in prison by their partner (termed “information explained misunderstanding”); and (3) participants who claimed that the information provided led to greater tolerance, either because it led to greater confidence in themselves, prepared them to be more patient, or incentivised them to help more (“information led to greater tolerance”). The remaining comments (20% of participants) were ambiguous, either because they did not provide an explanation, made tangential comments not related to the question, or ignored the question altogether.

**Table 4:** Frequency and distribution of coded statements from participants about the information received from the AC.

	No. of coded statements			Kruskall-
	(% of participants)			Wallis
		Dyslexia-	Autism-
	Control	disclosure	disclosure
	(*n* = 80)	(*n* = 83)	(*n* = 92)	H
Information redundant	37 (46%)	33 (40%)	28 (30%)	3.456
Information explained misunderstanding	4 (5%)	8 (10%)	12 (13%)	3.137
Information led to greater tolerance	14 (18%)	23 (28%)	44 (48%)	12.169^∗^
Ambiguous statements	26 (33%)	22 (27%)	15 (16%)	1.642

*^∗^*p*-value < 0.05. *Post hoc* pairwise comparisons with Bonferroni correction showed a significant difference only between participants in the autism condition and the control condition (*p* = 0.002).*

**FIGURE 2:**
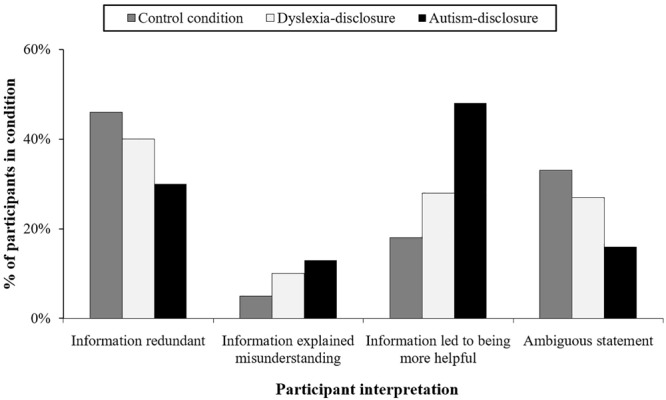
Comparison of participants’ perceptions about the information provided by the AC.

Table 4 and Figure 2 summarize the frequency and distributions of the coded statements. It highlights a trend in which participants in the autism-disclosure group were less likely to see the information they received from the AC as redundant and significantly more likely to claim that it led to greater tolerance in comparison with the control group (autism-disclosure group = 48%, control group = 18%). The dyslexia-disclosure group showed a similar trend but it was not significant compared with the control-disclosure group.

The finding that participants in the autism condition are significantly more likely to report that the AC information led to greater tolerance compares with increased perceptions of behaving in a more helpful way (reported above). It is also noticeable that diagnostic disclosure aids interpretations about the misunderstanding experienced, with 13% of participants in the autism condition linking the diagnosis to the misunderstanding compared with only 5% of participants in the control condition, although this is not significant at α < 0.05. Thus diagnostic disclosure of autism leads to increased perceptions of tolerance and increased likelihood that it will help to explain the misunderstanding experienced in the game. However, we also found that the same disclosure of a diagnosis can potentially have the opposite effects, being perceived as redundant information. Table 5 illustrates the range of statements provided by participants.

**Table 5:** Illustrative examples of reasons provided by participants for finding the information useful.

	Illustrative example		
Category	Control	Dyslexia-disclosure	Autism-disclosure
Information redundant	The information was so generic as to be useless in trying to formulate a strategy	They stated they were dyslexic, but I didn’t find that to be particularly useful information. It tells me nothing of their ability to perform the task	They just said they were autistic, which wasn’t really relevant
	While I understood what they was trying to share it was not useful to me	I guess it was useful but I’m not sure what being dyslexic has to do with rolling a ball through a maze	I did not know how to alter my playstyle through the information they shared with me, such as them being autistic
Information explained misunderstanding	They said they were not good at multitasking, which may explain why they did not notice my gate could have been unlocked	My partner shared they were dyslexic and had trouble with multiple tasks, and that might have explained why during one game they navigated through half the maze but failed to rescue me from the prison?	My partner explained that he was autistic which made it difficult for him to multi-task which explained why perhaps he forgot to do things in one of the games
	A little, he said he wasn’t good at multi-tasking (probably why he forgot to let me out as soon as he was able to) but he was good at maneuvering the ball	It tempered my frustration when my partner forgot to let me out of prison in one of the games	It was useful because I can somewhat understand how they performed the way that they did
Information led to greater tolerance	They told me they were good at steering, which they were, and they told me they weren’t great at multitasking which I had in mind when they forgot to free me until the end	It allowed me to be more empathic to the person controlling the other ball, and told me I didn’t need to worry about their control of the ball	He said as he was autistic he was not very good at focusing on multiple tasks at once, which made me more patient when I was locked in prison and only he could move
	I think I had left him confident and calm, so that we could play this game at our best	they said they were dyslexic but still good at following orders, I was ready to give them a bit more help of needed but they navigated just fine	They mentioned that they struggled to juggle multiple tasks at once due to suffering with autism. This was useful to know and was evident in some of the games that we played. As a result I tried to work as quickly as I could to complete my sections of the games, in order to compensate for times where my partner may have struggled to cope with multiple scenarios in the game

Thus, although disclosing a diagnosis of autism generally results in more positive perception (seeing disclosure from the AC as useful, perceiving oneself to be more helpful) it can also lead to negative perceptions (seeing the disclosure as redundant and useless). These differing reactions help to explain why disclosing a diagnosis may arouse anxiety for autistic people, because it is a cost-benefit decision which autistic people in particular may find especially hard to evaluate given that it would require a nuanced social reading of others. Moreover, the lack of an association between participants perceiving themselves to be helpful and helpful behavior suggests a bias in overestimating one’s own prosocial behavior toward autistic people.

## Discussion

The first research aim (RQ1) was to create a simulated interaction for exploring the psychological effects of labels exposed to different groups of participants. Dyad3D, combined with a post-game questionnaire, proved to be a very efficient means of gathering simulated interactional data. Participants found the interaction highly believable, with an average quality of deception rating of 3.83 out of 5, with only 9 participants (3.4% of participants passing all other inclusion/exclusion criteria) explicitly expressing doubt about whether the AC was human. All sampled participants also made attributions of intentionality, including mental/emotional states when describing the behavior of the AC showing that participants were psychologically orientated to the AC as another human player. The nature of the deception was also successful with participants providing feedback that it was efficient, believable, and enjoyable. Thus Dyad3D was broadly successful in creating an efficient, believable and controlled interaction that could be used to generate insights about the differential effects labels produce on social perception and behavior, although there remains scope for improvement especially in terms of introducing ecological complexity into the task (discussed further below). Further studies are required to help explore the viability of the method, thus a key contribution of the present study is to make the source code for the game freely available at: https://bitbucket.org/ enghoff/dyad3d.

The second research aim examined the effects of disclosing a diagnosis and was split into two parts. RQ2a compared self-reported data in the post-game questionnaire with behavior recorded in the game. Our findings concur with existing reports that the label of autism has a broad positive effect on social perception (Sasson et al., 2017), resulting in higher perceptions of intelligence of the AC, and perceiving information communicated by the AC as having more utility. However, there is also evidence to suggest that such effects are temporary, rather than enduring. Although participants find diagnostic disclosure about autism useful, they are less likely to believe it impacts their own ability to provide help in the task. In addition, there was no significant association between participants who believed that the AC diagnostic disclosure made them more helpful compared to whether they were actually helpful during the game. These findings help to explain why diagnostic disclosure, despite enhancing social perceptions by others, can still result in negative discrimination in terms of behavior as reported by autistic people themselves (Davidson and Henderson, 2010; Powell and Acker, 2016; Treweek et al., 2018).

RQ2b further highlighted why diagnostic disclosure is not straightforward, since there were varied reactions toward the information supplied by the AC. Although most participants found the information to be useful, they were less likely to articulate why. Many participants felt that the diagnosis of autism explained the misunderstanding experienced in the game, yet a smaller number of participants also felt that the diagnostic disclosure was redundant information. These varied reactions highlight why diagnostic disclosure is a risky decision for autistic people. The label of autism can ameliorate confusion associated with a misunderstanding, but it can also potentially exasperate underlying frustrations depending on the cognitive frame of the perceiver.

These findings contribute to understanding the *double empathy problem*, a term used to describe the differences in mutual understanding which arise between autistic and non-autistic people on account of their different dispositional outlooks (Milton, 2012; Milton et al., 2018). Interactions between autistic and non-autistic people are subject to bias, such as higher perceptions of awkwardness associated with autistic facial expressions (Faso et al., 2014; Brewer et al., 2016), or non-autistic people negating autistic social achievements in light of their diagnostic label (Heasman and Gillespie, 2018b). While to date research has focussed on how the label of autism affects the way autistic people are seen by others, the contribution of this study is to highlight that the label of autism also affects how others see themselves, specifically that they see themselves as more helpful than they actually are. Over-estimating one’s own helpfulness is understandable, since it protects the positive identity of the perceiver and brings their self-perception into line with their ideal Self as presented in the research. However it could lead to seeing less validity in the claims made by autistic people that such efforts toward them are not helpful; or, due to the paradoxical effects of helping (or even perceiving that one is being helpful: Gillespie and Hald, 2017), this bias may even lead to expectations that autistic people should be grateful.

Potential evidence of a “helping bias,” where participants perceive themselves to be more helpful toward autistic people than they actually are, has a number of real-world implications for autistic people. In a caregiving context, such findings align with other research on communicative disabilities where caregivers, and care-receivers have differing ideas about the levels of support being provided in the relationship (Goodwin, 2004; Moore and Gillespie, 2014). A helping bias would mean that caregivers are potentially less responsive to providing support if such support is seen by caregivers as surplus to the caregiving efforts already made. Within the context of employment, adjustments to the workplace can have a great impact on accessibility for autistic employees (Baldwin et al., 2014) and in many countries (e.g., the United Kingdom) it is a legal requirement to make reasonable adjustments for people with disabilities. However, the extent to which adjustments are seen as reasonable is subjective from the perspective of line-managers and HR professionals; a helping bias may be a contributory factor in explaining reports about why adjustments are rejected (Heasman, 2017), because managers, and HR professionals may already believe they are providing adequate support.

The study therefore highlights that diagnostic disclosure remains a risky decision for autistic people. The label of autism can lead to some improved perceptions of intelligence but also create additional mistaken beliefs about how helpful one actually is toward the autistic individual, which could become problematic in future interactions where levels of support are negotiated. Further research is required to understand how diagnostic disclosure can lead to more consistent and sustained positive effects on social perception and behavior. In particular, the relationship between autism knowledge and the psychological effects of disclosing a diagnosis of autism is receiving increasing attention (Gillespie-Lynch et al., 2015; Sasson and Morrison, 2017; Crane et al., 2018). It is possible that such effects may be associated with increased self-awareness of one’s own taken-for-granted assumptions toward others with a diagnosis, which raises the question as to whether knowledge of the effects of a helping bias could potentially serve to correct the effects of such a bias? It also remains to be seen whether these effects would be observed in interactions between autistic people, which have rarely been studied but have been shown to exhibit more complimentary features of interaction (Heasman and Gillespie, 2018a). Additionally, beyond those that perceived themselves to be helpful, future research should also explore further lay understanding of autism, since some participants reported that the diagnostic disclosure was irrelevant information for the purposes of collaboration.

In summary, this study has helped to illustrate both positive and negative discrimination resulting from a diagnostic disclosure of autism, with social perception more favorable than actual social behavior. Future research using simulated interactions can further differentiate the factors affecting social perception and behavior of non-autistic people toward autistic people, and in doing so potentially evolve the current design into an intervention for correcting biases that contribute to the double empathy problem. A central contribution of the study is therefore to make the source code for Dyad3D freely available. The study is reproducible and opens up the possibility for future studies to implement more ecological features into the game (e.g., varying the form in which the stimulus is presented which has been shown to shape social perceptions: Sasson et al., 2017), and to improve the sensitivity and diversity of behavioral measures.

## Limitations

Limitations pertaining to RQ1, the viability of the deception, stem from the use of a computer-mediated task which is contingent on people’s ability to interact with others via a computer interface. While this provides a means for replicating the experience of an interaction efficiently, it also raises questions about the validity of the interaction, because the identity of the AC is not open for questioning. The internet, and the development of networked virtual worlds, have created multiple opportunities for the same individual to present their identity in different ways (Gillespie et al., 2018) and consequently people are aware that virtual interactions may be risky and not authentic (Evans, 2012). Thus there may be levels of doubt associated with the authenticity of the interaction studied even if it is not explicitly mentioned or reported by participants when asked to rate the quality of deception. Moreover, such doubts may be furthered because of a lack of ability to see, hear or verbally interact with the AC which means such concerns about validity cannot be questioned. This means that there remains a question about the generalizability of such findings to other ecological contexts. It is possible, for example, that other modalities of interaction such as body language and physical appearance could alter whether positive or negative aspects of a diagnostic label become salient in social perception and behavior. As discussed previously, we deliberately limited the modalities of interaction in order to explore the viability of the process before iteratively introducing future ecological elements. Yet enhancing the strengths of the deception may itself be achieved through the introduction of further interactivity such as the ability to send messages in the game (which has since been added as a configurable option to the current game setup). Further structural improvements are also possible, such as improving collaborative basis of the game (e.g., the player and AC could share resources or empower each other’s abilities), or by changing the way in which the stimulus is presented (e.g., a pre-recorded webcam of the “online” player).

Limitations pertaining to RQ2a, which compares self-report and behavioral data, highlight concerns about the social aspects of the game and what it reveals about interpersonal dynamics. In the present study we deliberately presented a minimalist situation to see what knowledge participants would import to the disclosure of a diagnosis, particularly since in real life discovering someone’s diagnosis may not always be associated with adequate auxiliary information about the diagnosis. Thus it was interesting to find that even in a predominantly cognitive task with minimal information about the diagnosis, disclosure resulted in positive evaluations of the ACs’ intelligence, and utility of information shared. However, in this context there is no reason not to be generous in social perceptions. Perceptions might change if the consequences were higher (e.g., job hiring) where there was a major investment in the outcome of the interaction.

Another challenge of the present design is that Dyad3D is a primarily goal-orientated activity which may supersede the social obligation to help one’s partner. Although a social component exists in the game in terms of freeing the other player from prison and having to share responsibility for opening different doors in the game between player and AC, it is still possible to play the game in a primarily strategic way. Revising structural aspects of the game and associated behavioral measures should therefore be explored to build a more ecologically valid understanding of social interaction. For example, there could be individual scores for each player which are then aggregated to form an overall score for the dyad. This could help boost an understanding of teamwork and collective identity. Likewise, there could be a reward system which benefits the dyad if close spatial proximity is maintained. No significant differences were observed in mean distance between players, mean time to complete levels, mean time between collecting gold cubes, mean keystrokes, or mean keystroke duration. These null results indicate that the significant behavioral differences observed should be interpreted with a degree caution and highlight room for improving the way behavioral measures operationalized in the game detect meaningful action.

Limitations pertaining to RQ2b, understanding the explanations provided by participants, highlight a potential bias in terms of memory retrieval. In-game activity may deplete attentional resources required to accurately report on one’s interaction, although there were no significant differences observed when comparing gaming experience with helpfulness or recognizing the information shared as useful. Moreover, Dyad3D used deliberately simple and intuitive input controls of the arrow keys, yet even this can place a demand on users not familiar with computers or navigation via keyboard inputs, which represents another potential distraction from accurate reporting. The ability to ostensibly exchange messages (i.e., a chatbot interface) could represent more valuable qualitative data, as instead of asking participants to report on the interaction in hindsight, one can observe their actual attempts at communication to build a social understanding of the situation. To help address this challenge, a configurable chat interface option has already been included in the existing source for the game.

Finally, within the protocol we examined success in terms of whether participants believed they were interacting with a human. However, deception arguably also involves a second component, which is whether participants believe in the diagnosis that was disclosed. In our data, no participants gave an indication that they did not believe in the diagnosis from their verbal explanations of the interaction, but further studies may want to explicitly ask this question post-deception reveal. Additionally, we did not assess levels of autism knowledge since there are conflicting findings about whether there is a relationship between knowledge and attitudes in understanding autism (White et al., 2016), and the cross-cultural bias of such tools has been questioned (Harrison et al., 2017). Nevertheless, further studies could benefit from implementing an autism knowledge questionnaire, particularly as it could help to understand more about why there are both positive, and negative aspects of discrimination associated with disclosure as observed in the present study.

## Ethics Statement

This study was carried out in accordance with the recommendations of The Research Ethics Code, The London School of Economics Research Ethics Committee with written informed consent from all subjects. All subjects gave written informed consent in accordance with the Declaration of Helsinki. The protocol was approved by the London School of Economics Research Ethics Committee (REC approval ref.: 000674).

## Author Contributions

BH designed and wrote the code for Dyad3D, recruited the participants, conducted the pilots, gathered and analyzed the main study data, and wrote the manuscript. AG provided the intellectual guidance in developing the research throughout, including through discussions, and commentary on aspects of the written manuscript.

## Conflict of Interest Statement

The authors declare that the research was conducted in the absence of any commercial or financial relationships that could be construed as a potential conflict of interest.
